# Culture, salience, and psychiatric diagnosis: exploring the concept of cultural congruence & its practical application

**DOI:** 10.1186/1747-5341-8-5

**Published:** 2013-07-16

**Authors:** Mohammed Abouelleil Rashed

**Affiliations:** 1 Department of Psychiatry, Division of Philosophy and Ethics of Mental Health, University of Pretoria, Pretoria, South Africa

**Keywords:** Cultural congruence, Cultural learning, Diagnosis, DSM, Ethnography, Experiential dispositions, Intentionality, Phenomenology, Psychiatry, Salience

## Abstract

**Introduction:**

Cultural congruence is the idea that to the extent a belief or experience is culturally shared it is not to feature in a diagnostic judgement, irrespective of its resemblance to psychiatric pathology. This rests on the argument that since deviation from norms is central to diagnosis, and since what counts as deviation is relative to context, assessing the degree of fit between mental states and cultural norms is crucial. Various problems beset the cultural congruence construct including impoverished definitions of culture as religious, national or ethnic group and of congruence as validation by that group. This article attempts to address these shortcomings to arrive at a cogent construct.

**Results:**

The article distinguishes symbolic from phenomenological conceptions of culture, the latter expanded upon through two sources: Husserl’s phenomenological analysis of background intentionality and neuropsychological literature on salience. It is argued that culture is not limited to symbolic presuppositions and shapes subjects’ experiential dispositions. This conception is deployed to re-examine the meaning of (in)congruence. The main argument is that a significant, since foundational, deviation from culture is not from a value or belief but from culturally-instilled experiential dispositions, in what is salient to an individual in a particular context.

**Conclusion:**

Applying the concept of cultural congruence must not be limited to assessing violations of the symbolic order and must consider alignment with or deviations from culturally-instilled experiential dispositions. By virtue of being foundational to a shared experience of the world, such dispositions are more accurate indicators of potential vulnerability. Notwithstanding problems of access and expertise, clinical practice should aim to accommodate this richer meaning of cultural congruence.

## Introduction

A certain insight has made some headway within psychiatry. In 1994, the Diagnostic and Statistical Manual of Mental Disorders (DSM-IV) stipulated that religious beliefs are not delusions if “ordinarily accepted by other members of the person’s culture or subculture” ([1], p. xxiv) and “hallucinations may … be a normal part of religious experience in certain cultural contexts” (p. 275). More recently, consultation papers for the DSM-5 [2] acknowledged that in some clinical encounters there will be “difficulty in making a diagnostic judgment, owing to a significant difference in cultural, religious, or socioeconomic background of clinician and patient”. In such cases, “when there is uncertainty about the match between culturally expressed symptoms and diagnostic criteria”, attention to cultural formulation is imperative.^a^ Given these caveats and advice, when clinicians encounter beliefs and forms of experience that recall psychiatric pathology they should not unreflectively base their judgement on their own turnip patch, whether this happens to be the norms encoded in psychiatric categories or their personal values and beliefs; what they should do is seek a relevant context as a basis for judgement.

All this sounds plausible. Some account of deviation from epistemic, social, ethical or other norms must be given – or rather is already implied – in the diagnosis of mental disorder. However, before one can establish if a deviation is clinically significant, it is important to judge whether it counts as a deviation in the first place, hence the need for a suitable context as a basis for judgement. The context intended here is not limited to the framework of the prospective patient and includes the individual’s sociocultural environment. The clinician needs to establish the presence or absence of ‘cultural congruence’, which is the degree of fit between mental states and cultural norms. This cannot be resolved, as indicated, solely by asking the patient, since what has (potentially) gone amiss, as judged provisionally, is his or her benchmark of what is normal or acceptable, and what is needed is an understanding of the context within which to further assess such a judgement. Clinicians do this on a daily basis; they do it implicitly through the process of diagnosis, particularly when there is an unquestioned assumption that patient and doctor share a cultural context. When suspicions arise that cultural differences might impair judgement, a benchmark other than the clinician’s becomes required.

There are, however, a number of conceptual and practical problems that undermine the usefulness of the cultural congruence construct. Elsewhere ([3], pp. 194–198) I presented a critique of the psychiatric approach to cultural congruence. I argued, first, that it operates with a deficient view of culture as consisting in bounded religious groups or ethnicities to which patients can be ‘assigned’. Second, it fails to do justice to congruence which is treated as mere validation: once a person’s reference group is identified, the opinion of representative (and sane) members of that group with respect to the person’s mental states is sought; the absence of validation supports the judgement that the mental states are ‘abnormal’. I suggested that culture should be understood as symbols and meanings in flux, and determining a person’s cultural reference points is not exhausted by presumed affiliation and requires an investigation of the myriad influences they are and have been exposed to (see also [4,5]). Further, understanding congruence as validation presupposes the existence of a coherent set of norms accessible to those whose opinion we seek and divorced from issues of power, commitments and aesthetics, an untenable assumption given people’s diverging priorities and concerns. Instead, I proposed, we should seek an independent study of the cultural epistemology – presuppositions about the limits of experience and sources of knowledge – as a basis for assessing mental states. Then it will be seen, to invoke an example I used in the paper, that while it might be normal for a Lakota Indian to experience the voice of a deceased ancestor, a white North American having a similar experience should evoke concern.

Recently (September 2012) in the context of a University of Glasgow project entitled ‘Debating the First Principles of Transcultural Psychiatry’,^b^ the authors discussed my proposal [3] for a refined concept of cultural congruence and raised a number of important issues. While generally sympathetic to my attempt, they objected that it “may still nonetheless reify culture into a static, independent entity” ([6], p. 2). This, according to Miller and colleagues involves “an alienation of culture from the agency of persons”. They asked:

Whatever the “epistemologies” of Native Americans and “white” North Americans (populations referred to by Rashed), were they really so bound to such presuppositions? [and it is not clear why a] white North American hearing the voice of the dead is having a pathological experience, while the Lakota is not. If it really is a matter of cultural congruence, why not introduce the white North American to some Lakota, or to some other white North American voice-of-the-dead hearers? ([7], p. 6).

These are concerns that go to the heart of the matter and raise a number of questions:

• What are the ‘presuppositions’ of a cultural context?

• Are individuals bound to cultural ‘presuppositions’?

• Are all deviations from cultural ‘presuppositions’ significant, i.e. indications of a problem or vulnerability with the subject?

• To what extent can these deviations be detected across cultural contexts and in clinical practice?

This paper attempts to address these questions to arrive at a conceptually sound and useful construct of cultural congruence. The term ‘presuppositions’ in the foregoing questions suggests something fundamental and basic about culture relative to which the subject’s mental states should be aligned and against which they can be assessed. Gaining some clarity on these issues requires an understanding of the concept of culture.

The paper begins with a first-person account of two complementary ways in which culture is acquired: knowledge and participation. Section two builds on this account by drawing a corresponding distinction between symbolic and phenomenological conceptions of culture. The latter is further developed through the articulation of the ways in which, through participation, socially acquired meanings and significances organise background intentionality. Culture is thus not limited to symbolic presuppositions, and extends to shaping subjects’ experiential dispositions. Section three employs this view of culture to address the four questions raised earlier. The main argument is that a significant, since foundational, deviation from culture is not from a value or a belief as such but from culturally instilled experiential dispositions, specifically in what is salient to an individual in a particular context. A cogent concept of cultural congruence should be sensitive to this distinction. Section four demonstrates how the revised concept of cultural congruence can be applied, first, in the ideal context of an extended, ethnographic research setting and, second, in the more restrictive setting of the clinic.

## A case study in cultural learning

Genuine cultural learning is a two-faceted process; it requires gaining *knowledge* of beliefs and symbols and *participation* in a social context, the latter essential for achieving shared perceptual attunement with the environment. The following first-person account draws this distinction and serves to highlight the indispensability of direct engagement to cultural learning, an idea that will arise later in the article as a potentially, if partially, surmountable obstacle to the application of the concept of cultural congruence in the clinic.

In 2009 and 2010 I spent several months conducting field-work in the Dakhla oasis, one of six oases in the Western desert of Egypt. A common phenomenon in the oasis, in fact in Egypt generally as well as other Mediterranean societies, is the ‘evil-eye’ (*‘ayn*). It is an innate capacity to do harm delivered unintentionally through a direct look when encountering abundance or beauty in situations that evoke genuine admiration and appreciation. The evil eye is closely related to envy (*‘hasad*): both result in harm, but with the latter this is brought about intentionally. An envious person harbours feelings of covetousness towards others’ possessions, health, beauty, or any object or attribute considered desirable. In practical usage envy also implies a wish on behalf of the envier, motivated by bitterness, to see the other person stripped of these positive attributes. On the other hand, the evil-eye may occur from a good, pious person, in the absence of any intention to cause harm. It may affect animals, plants, material possessions and human beings. People regularly explain misfortune – for example if a tree dies or a well dries – by saying that a person who visited recently had given a ‘bad eye’. While it is generally understood that a person may not be able to control the evil-eye, there are certain protective prayers that should be invoked when abundance and beauty are encountered.

In the first few weeks at the oasis I was able to thoroughly learn beliefs concerning envy and the evil-eye. Shortly after, I realised that applying this knowledge in context was not as straightforward as I had thought. I was visiting the livestock shed of a Qur’anic healer I had been working with at the time. They had three healthy, well-fed cows, one of which was pregnant, in addition to several chickens, goats, and a couple of sheep. As we entered, they started the usual invocations when seeing abundance and the healer sprinkled over the pregnant cow some blessed-water he had previously prepared. I found myself caught in a double-bind. Not to praise what I saw is rude and may be construed as jealousy, yet to praise too much is to seem disingenuous or, even worse, an attempt to mask envy. Not to look is socially unacceptable and also rude, yet staring might be construed as ‘giving the eye’. I felt palpable anxiety in trying to negotiate my response, consciously and delicately: praising sincerely yet in a measured way, looking but not staring, and sharing in the invocations they were repeating. Knowledge of the cultural concepts of envy and the evil-eye in a relevant context had impacted on my responses.

In time I found myself more comfortable at negotiating these situations. In fact, something else had occurred, a subtle yet significant shift in my engagement with the environment. Before, I was concerned with applying the knowledge I had gained in order to understand why people around me were behaving in this way and to know how I should behave. Now, there was a natural sense of attunement with the same environment which earlier I had been at pains to interpret. And this was not a matter of speed of interpretation, of being more adept at applying knowledge to a situation in order to understand it. This was now *my* environment of action, which did not emerge as an interpretive conundrum but as a medium imbued with significances and affordances. It was then that the experience of a pregnant cow was transformed from something which hitherto was only relevant in so far as thinking about it aided the interpretation of social situations, to something that was naturally significant, compelling me to act in certain ways. At that point I considered myself to have achieved genuine cultural learning in that specific aspect of the sociocultural environment of this community. And this occurred through a two faceted process: *knowledge* of beliefs and symbols, and *participation* in a social context.

## The concept of culture

The concept of culture is among the more complex academic concepts. It has multiple meanings and uses within and across different disciplines as well as the vernacular in which it is a widely used term. Further, the concept has changed and developed through the decades in quite radical ways, and some of its earlier conceptions, now, to us, may appear surprisingly ethnocentric if not racist.^c^ It is rather important then to narrow down on those aspects of culture that are relevant to the problem areas this paper tackles. The preceding ‘case study in cultural learning’ goes some way towards this goal. By looking at how culture is acquired we have come to identify the ways in which shared meanings and significances condition subjectivity and influence behaviour. This coheres with the central concern of this paper, which is to understand the relations between culture and subjectivity, when these relations can be said to be problematic, and whether or not it is possible to detect this.

As I have come to learn through my experience in Dakhla, cultural learning requires developing knowledge of symbols and beliefs such that one is able to share a cognitive understanding of social situations. It also requires participation in the ebb and flow of a social context in order to be attuned to the environment and be moved to feel and act in an immediate and natural way. These two facets of cultural learning correspond to two views of culture which have developed over the course of the second half of the 20th century: the symbolic and the phenomenological. These views may be usefully thought to differ with regards to the answer to this question: In what ways does culture – understood as socially acquired meanings and significances – condition subjective experience? Symbolic views emphasise the act of interpretation and meaning-giving, while phenomenological views, in addition, of course, to recognising the symbolic order, highlight more passive, prereflective modes of engagement with the world. In the remainder of this section, this distinction is further highlighted and the phenomenological view of culture is expanded upon through the concepts of intentionality and salience. The emerging account will provide a framework over which a more fruitful conceptualisation of congruence can be based.

### Symbolic & phenomenological conceptions of culture

Throughout the Enlightenment, culture stood in opposition to the body, and, more generally, to nature (cf. [5]). This opposition was replicated in the emerging discipline of anthropology and by the first half of the twentieth century culture came to denote more narrowly the “conceptual and linguistic dimensions of human existence to the exclusion of somatic, sensory, and biological dimensions” ([8], p. 18). Leslie White, writing in 1959, took culture to mean the “extrasomatic*,* temporal continuum of things and events dependent upon symboling” ([9], p. 3). Symbolic views were carried forward by Clifford Geertz’s influential conception of culture as an

historically transmitted pattern of meanings embodied in symbols, a system of inherited conceptions expressed in symbolic forms by means of which men communicate, perpetuate, and develop their knowledge about and attitudes towards life ([10], p. 89).

Here, culture is an “interworked system(s) of construable signs” that provide the context within which behaviour and social events can be understood among members of a community ([10], p. 14). This is evident, for instance, in the importance of grasping the phenomena of the evil-eye and envy. The kind of context Geertz is after is the subtle understanding that enables one to interpret an eye twitch as a wink and not only an eye lid contraction. It is the ethnographer’s task to understand such a system of significations if she is to gain access to the conceptual world where subjects live ([10], p. 24). In a more recent formulation of the symbolic view, culture has been described as “shared symbols and meanings that people create in the process of social interaction,” where it functions as a resource that shapes experience, interpretation, and action, and “orients people in their ways of feeling, thinking, and being in the world” ([11], p. 5). Cultural symbols and signs invest the environment with meaning and through being shared permit intersubjective understanding and communication.

Symbolic approaches to culture have been criticized as narrow and incomplete, and for eliminating the possibility of meaning and engagement that precede representation. They privilege culture and mind over biology and body; culture begins where biology ends and the mind becomes a representational machine engaged in inferential relations with objects in the world, including its own body, and adopting the cultural framework to thematise and give meaning to what are culturally-neutral experiences. Against this view, some anthropologists (e.g. [8,12-14]) appealed to the phenomenological tradition in philosophy to argue for a concept of culture which recognises that socially acquired meanings and significances are evident in our natural attunement with the world. In what follows I will attempt to locate culture phenomenologically by exploring aspects of the concept of intentionality and its relation to salience. In particular, I argue that far from being limited to reflective modes of engagement, the influence of culture can be seen in the organisation of background intentionality, specifically in the automatic attentive orientation by virtue of which certain aspects of the environment are imbued with more salience than others.

### Intentionality

Intentionality is a basic characteristic of consciousness. Consciousness is always consciousness of something whether real or imagined. The memory of a friend, a hallucinated dragon, and a perceived car are all intentional experiences with the friend, dragon, and car being the respective intentional objects. In *Logical Investigations* Husserl ([1913] [15]) identified two inseparable aspects of intentional experiences: intentional matter and intentional quality. The former specifies the object intended and the perspective through which it is apprehended given one’s background and context. Perception, thus, is informed by “valences, feelings, past experiences, and frameworks of reference and interest” ([16], p. 115). The intentional quality, on the other hand, specifies the *type* of experience; whether the object is remembered, judged, doubted, desired, denied, or feared (cf. [17], p. 121).

The intentionality just outlined is active and thematic; it discloses objects in the world and takes up positions and judgements towards them. It is founded upon more basic and passive forms of background intentionality. In *Experience and Judgement* ([1948] [18]) Husserl writes:

The activity of perception, the perceptive orientation toward particular objects, their contemplation and explication, is already an *active performance of the ego.* As such, it presupposes that something is already pregiven to us, which we can turn toward in perception. And it is not mere particular objects, isolated by themselves, which are thus pregiven but always a *field* of pregivenness, from which a particular stands out and, so to speak, “excites us” to perception and perceptive contemplation ([18], p. 72).

Husserl then proceeds to provide a phenomenological explication of the passive process whereby aspects of the intentional background experience stand out and command the attention of the ego. He distinguishes two stages in this process: the first is a tendency preceding the *cogito*; a passive, prereflective stage in which various stimuli obtrude upon the ego accompanied by a tendency on behalf of the ego to “give way” and be attracted as it is in its nature ([18], p. 78). The second stage is the compliance of the ego with the initial tendency and the turning of the ego towards the object; a basic state of receptivity in which the *cogito* becomes active and may proceed towards reflective and thematic forms of intentionality. In this state the ego has received “what is pregiven to it through the affecting stimuli” ([18], p. 79).

What determines which particulars “stand out” in the background experience prior to the activity of the ego? How are certain particulars able to affect the ego, i.e. to “stand out from the environment, which is always copresent…[and] attract interest to oneself, possibly interest in cognition” ([18], p. 30)? Husserl employs the example of a field of sensuous data as the simplest model to demonstrate the way in which the passive field of perception possesses prominences and particularities. The initial synthesis of a field of sensuous data occurs though elements that contrast with others and are raised to prominence. Homogeneity and heterogeneity both within and across different fields, e.g. visual and auditory, allow for the articulation of the field into prominences. Through further processes of *associative synthesis* – in which like and unlike elements recall or “call attention” to each other – groups arise in the field and certain members begin to emerge from a homogenous background ([18], pp. 72–75).

Those elements which stand-out in a field, through their intensity, begin to exert a stimulus or “obtrude” upon the ego. Husserl gives the example of a noise or a colour which on the basis of its intensity may exert a powerful or weak stimulus, and may initiate a turning-toward of the ego. But Husserl is not limiting his phenomenological explication to sensuous data. He indicates that associative synthesis “holds in the same way for all data” ([18], p. 75), and that thoughts and desires, and not only sounds or colours, can obtrude upon the ego from the background with varying degrees of insistence ([18], p. 76). However, as he recognises, the mode of “coming-to-prominence” will vary: with sensuous data this will be determined by contrasts and *qualitative* discontinuities which cannot be present with non-sensuous data. Nevertheless, he writes, “among the different obscure movements of thought which stir us, one thought … stands out from all the rest and has a sensitive effect on the ego, as it, so to speak, forces itself against the ego” ([18], p. 77).

In summary, obtrusion upon the ego is a function of a discontinuity in a field of perception which arises on the basis of the insistence and intensity of stimuli whether of a sensuous or non-sensuous nature. This raises the question: what other kinds of discontinuities are there? A helpful distinction can be found in neuropsychological research on salience and attention. Another way for referring to the “standing out” or “coming-to-prominence” of aspects of a perceptual field is to say that those aspects are more salient than others; i.e. relevant or important.

### Salience

Upon encountering a visual field, the subject weighs, so to speak, the multitude of data presented. On the basis of this a “salience map” is constructed, which is a “representation of the environment that weighs every input by its local feature contrast and its current behavioural relevance” ([19], p. 430). Only those elements deemed salient become targets for saccadic eye movements and further allocation of attentional resources. Salience research recognises two influences on the process of construction of a “salience map” (see [20-22]). The first are “bottom-up” influences concerned with the properties of the stimulus itself such as colour, size, luminance and contrast. The second are “top-down” attentional influences concerning “prior expectation, memory or emotional association” ([22], p. 902), and where the “observer’s expectations or intentions influence the allocation of attention” ([20], p. 107). It is mainly the first set of influences that are addressed by Husserl’s phenomenological explication, influences constitutive of prominences in a field of perception. But what about “top-down” influences?

“Top-down” influences, as suggested by salience research, reflect the state of the subject as opposed to the state of the stimulus. They thus reflect, as indicated, purposes, memory and expectations. This might seem to suggest that such influences involve an active accomplishment of the ego and thus do not qualify as prereflective influences on the constitution of background intentionality. To be sure such influences can be voluntary; they may involve an act of will in attending to an object or constructing a theme, but they need not always be. It is possible to conceive of prereflective yet purposeful constructions of salience, arguably through positing unconscious drives or repressed memories that direct attention. In addition, and this is the concern here, it may also be possible to conceive of prereflective constructions of salience that are culturally, rather than individually, determined. To expand on this point, consider the connection between cognitive processing styles and perception.

Recent studies demonstrated cultural differences in styles of cognitive processing among ‘Westerners’ and ‘East Asians’. While the former are more likely to attend to focal objects, engaging in categorisation and rule discovery, the latter emphasise context and are more likely to attend to a “broader perceptual and conceptual field, noticing relationships and changes and grouping objects based on family resemblance rather than category membership” ([23], p. 11163; see also [24]). It has been hypothesised that these cultural differences in cognitive style arise on the basis of perceptual differences in the way attention is automatically allocated via saccadic eye movements and fixation to varying aspects of the environment ([23], p. 11169). Chua and colleagues [25] examined this hypothesis by presenting pictures with a single foreground object and a realistic background to American and Chinese subjects and monitoring their eye movements via a tracking device. They found that Chinese subjects looked more at the background and were less able to correctly identify old foreground objects that were presented in a new background. The American subjects looked at foreground objects sooner and longer than the Chinese subjects. These results suggest that there are cultural differences in the allocation of attentional resources at the primary level of visual memory, and reveals a possible reason for higher-level cognitive differences in holistic versus focal processing of information ([25], pp. 12631–12633).

What we have here are culturally determined top-down influences on the construction of salience that occur prior to the objectifying activity of the ego, complementing bottom-up (stimulus) influences. How do these cultural differences arise? A possible answer is through socialisation; by virtue of immersion in a specific sociocultural context, an individual comes to internalise not only frameworks of interpretation but also the basic and automatic attentive orientation to the environment by virtue of which certain aspects are imbued with more salience than others. And what purpose do these cultural differences reflect, i.e. why do ‘Westerners’ attend more to the foreground and ‘East Asians’ to the background? The answer put forward by Nisbett and Masuda [23] and Chua and colleagues [25] is that differences in visual memory are a function of differences in sociocultural conditions. The idea is that on the basis of material, geographic and other factors societies tend to develop distinctive social structures and practices. ‘East Asian’ societies emphasise hierarchies, role relations and possess complex social networks. In such a social environment, it is more important that social actors attend to context and inter-relations than it is to adopt an instrumental attitude to objects defined by individual goals, and the converse holds for ‘Western’ societies. In any case, irrespective of the explanation of *why* these cultural differences exist, the evidence suggests that they do, and that they influence the automatic, top-down allocation of attention prior to the reflective activity of the ego.

### Comments

In summary, background intentionality, understood as salience, betrays evidence of cultural organisation prior to the reflective activity of the ego. What we see – and not just how we see it – is influenced by culture, and this precedes personal and active judgements and positions. Merleau-Ponty in *Phenomenology of Perception* ([1949] [26]) identifies this insight when he draws a distinction between

intentionality of act, which is that of our judgements and of those occasions when we voluntarily take up a position … and operative intentionality, or that which produces the natural and antepredicative unity of the world and of our life, being apparent in our desires, our evaluations and in the landscape we see, more clearly than in objective knowledge, and furnishing the text which our knowledge tries to translate into precise language ([26], p. xviii).

The “landscape we see”, as Merleau-Ponty refers to it, varies, as the studies above suggest, relative to what are quite broad sociocultural categorisations. Individuals are not only socialised into broad or primary social contexts but also into local or secondary ones, as we find with certain professions and activities. For instance, to become a farmer one not only requires *knowledge* of soil types, seasonal variations, fertilisation, and so on; one comes to develop subtle *perceptual* discriminations of what to most people is simply a patch of land. The patch of land offers a different set of affordances, or opportunities for action, to the farmer than to the uninitiated. This is partly on the basis of the way in which otherwise innocuous or unnoticed aspects of the field of perception come to prominence and emerge as relevant and important; i.e. as salient. We can thus talk of culture in terms of a broader or narrower world of common-sense, knowledge and practice. In both senses of culture, we find that socially acquired meanings and significances condition prereflective subjective experience.

Finally, I would like to re-reflect on the ‘case study in cultural learning’ presented in the previous section. If we understand culture as symbols and meanings that provide the context within which behaviour is understood, then my knowledge of the evil-eye and envy enabled me to interpret and respond to a complex social situation whose subtleties would otherwise have been lost on me. If we understand culture in the complementary phenomenological sense of socially acquired significances evident in the organisation of background intentionality, then my immersion and participation in the social context of Dakhla reordered my experience of the environment, aspects of which began to assume a hitherto absent sense of salience.

## Cultural congruence

Culture consists in shared meanings and significances that condition subjective experience, influence behaviour and permit individuals to share a cognitive and experiential understanding of the world. These influences can be apprehended at the symbolic and phenomenological levels, with the specific nature of each level articulated in the foregoing analysis. In what follows, this view of culture will be employed to address the questions concerning cultural congruence which this paper started with. To begin, the questions will be answered through adopting the symbolic view of culture. In doing so, the limitations of this view will become apparent and the need for incorporating phenomenological views evident.

### What are the ‘presuppositions’ of a cultural context?

If we adopt the symbolic view of culture, then presuppositions, or assumptions, in this context are beliefs about the world that are considered basic and fundamental. To name a few, there are presuppositions about the cosmos; the ultimate nature of reality, the (in)existence of a supernatural world, the place of human beings in it, the possibility of being influenced by outside forces and to influence others in like fashion; the nature of social interaction and social norms; epistemological assumptions about the limits of experience and accepted or valued sources of knowledge; questions of value and aesthetics, of what people implicitly take as worthy or important.

### Are individuals bound to cultural ‘presuppositions’?

What does ‘bound’ mean in this context? One way of understanding being ‘bound’ to cultural presuppositions is to say that certain frameworks of belief and value *apply* to the person and in such a way that violating them is a reason to judge that person's capacities. But whence do they apply?

‘Apply’ can be understood in the sense of ethically and legally binding. For example liberal beliefs and values concerning equality, tolerance and the right for freedom of expression can be said to be ethically and legally binding for individuals who exist in modern multi-cultural societies. Violating such principles and values can be judged wrong and in some cases illegal. Similarly, in some societies belief in God, religious doctrine and a supernatural world can be said to apply to individuals who live in these communities, with various sorts of sanctions for those who reject this. (The question of which, if any, framework *should* apply is a separate one and not relevant to the issue addressed here.) However, what we are after is not a contingent sense in which cultural presuppositions can be said to ‘apply’ to a person but rather something that is called for by a certain expectation of learning, such as dictated by the nature of socialisation.

Consider this from the narrower view of professional socialisation. If I have made a decision to become a farmer then it is plausible to assume that, in time, beliefs and rules pertaining to farming would apply to me such that they can be employed to ascertain whether I am a good farmer or not. This is just called for by my commitment to farming, and the *expectation* that in a year I must have learned something of it. If we extend this analogy to socialisation in general we can say that through immersion in a particular sociocultural context, an individual is expected to have grasped and to be able to apply frameworks of interpretation, to become part of that normality, so to speak. Failure to do so raises questions about the reasons behind this, and doubting the subject’s capacities arises as one, but not the only, possibility. A central difficulty here concerns the demarcation of the sociocultural context of relevance to the individual. With farming and professional socialisation in general this is relatively straightforward, but with the broader context, specifying those influences which the person is expected to have internalised and mastered is far from straightforward. This problem, the problem of demarcation, will be addressed in the final section of this paper.

### Are all deviations from cultural ‘presuppositions’ significant, i.e. indications of a problem or vulnerability with the subject?

The violation of cultural presuppositions, as indicated, invites speculation; why has this violation occurred? Is it significant or not? Consider this, again, from the view of professional socialisation. If I have been trying to become a farmer for a year and at the end of it I am still unable to understand the basics of agriculture then it is fair to say that I am a failed farmer; that my initiation or socialisation into ‘farmhood’ has been unsuccessful. This may be due to (i) a *problem* with my capacities. But even if I fail despite trying, other explanations present themselves: maybe (ii) my teacher was not sufficiently knowledgeable or intentionally made sure I would fail; or maybe (iii) I had creatively appropriated and developed knowledge of farming in a way that exceeded the understanding of my teacher. Transporting this analogy to the arena of mental health, we have a number of candidate explanations for the violation of norms and beliefs that lies at the heart of social definitions of ‘deviance’: (i) *dysfunctional* capacities^d^ (‘mental disorder’); (ii) *normal* capacities (apparent violation due to neglect or social discrimination); (iii) *normal/highly developed* capacities (creativity). Only the first explanation seems to be of health-related interest while the latter two we may call ‘benign violations’. The question that arises here is this: how can we distinguish benign violations from those that are of provisional health-related interest?

### Distinguishing benign and significant deviations from culture

As indicated, it is the first explanation of deviation from cultural presuppositions – dysfunctional capacities – that suggests a problem or vulnerability with the subject, and which a cogent concept of cultural congruence should aid us in picking out. However, if we remain with symbolic views of culture, then the concept of cultural congruence can only tell us if norms had been broken or beliefs violated, but cannot tell us whether these violations are significant or not in the terms outlined here; i.e. whether they are benign or otherwise.^e^ And the problem goes the other way too; not only is the concept liable to produce false positives (benign violations taken as significant violations), there is also a risk of false negatives where a real vulnerability may be missed because at the level of norms, values and beliefs there is no violation with respect to cultural presuppositions. But here I am begging the question: I am invoking a vulnerability that is independent of the violation of sociocultural norms, and that can be present in the absence of obvious outward manifestations. To illuminate what is intended it is necessary to incorporate phenomenological understandings into the concept of cultural congruence.

As argued in the preceding section, background intentionality betrays cultural organisation evident in the prereflective construction of salience. In this sense a significant deviation from culture would not be from a norm, value or belief per se but from such culturally instilled experiential dispositions, specifically in what is salient to an individual in a certain context. Such dispositions permit a shared experience of the world that is prereflective and foundational to further cognitive constructions. Deviating from these dispositions constitutes a vulnerability that *may* lead to a gradual idiosyncratic and subjective rendering of shared reality. The harm that may then ensue is on the basis of the gradual detachment from shared reality evident in the eventual formation of non-consensual beliefs about the world and the negative repercussions (social and otherwise) that tend to follow from that. Thus it is not the vulnerability itself that is the problem but its consequences. This might seem to suggest that what really matters is not the vulnerability but the beliefs that arise on its basis, precluding the need to identify it. This is true in terms of the harm that ensues; however, the conceptual distinction that needs to be drawn here is between non-consensual (and potentially harmful) beliefs that are benign violations from non-consensual (and potentially harmful) beliefs as well as consensual (and non-harmful) beliefs that are *underpinned by a foundational vulnerability*.^f^

A potential objection here is that within the category ‘states underpinned by a foundational vulnerability’ it will not be possible to distinguish states of creativity from other health-connected states. And given that the distinction was intended to identify those cases that are, provisionally, of health-related interest, then it fails on this account. The objection, thus, will be that at least some creative states are not only about the appropriation of existing symbols in novel ways but are underpinned by a radical, if circumscribed, deviation from shared experiential dispositions. These are elaborated in ways that are accepted or even admired by others. In responding to this objection it is important to note that the vulnerability intended – as the term *vulnerability* implies – is a potentiality that may not necessarily lead to harm in the form of an idiosyncratic rendering of shared reality. The idea is that this vulnerability permits both positive and negative outcomes, in the case of the former it may even be described as a ‘gift’. It is sufficient for the distinction drawn here that the vulnerability (or gift) is delineated irrespective of what it may lead to. Indeed, it could be further argued that detecting such experiential underpinnings may allow the provision of support to willing individuals such that they develop their vulnerability/gift in ways that promote rather than hinder their wellbeing.

Given the distinction drawn here, we can now turn to potential application. How can we identify foundational vulnerabilities – or deviations from shared experiential dispositions – in practice? And how is this to be accomplished without relying on the violation of cultural presuppositions which, as argued, cut across this vulnerability? The key is to attend to it directly. The following section illustrates how this can be done, first, through the context of participant observation as provided for in prolonged ethnographic research and engagement with subjects and, second, in the context of the clinic.

## Cultural congruence in practice

### In the ‘field’

Ahmed^g^ is a young man I met during my research. After enduring a humiliating beating at the hands of his school teacher before the entire morning assembly he decided to stop attending school. This was only a few weeks before his finals and his father urged him to return. He was particularly angered at his father’s refusal to confront the teacher and secure his dignity. He left home and isolated himself in a deserted mud-brick house owned by his family; he was too embarrassed to face the village following his humiliation. His mother was intensely saddened by her son’s sudden change in fortune, and suspected that he was subject to envy or perhaps magic, a common explanation in the oasis for these kinds of situations. The presence of magic was more or less confirmed when she found a small, square black bundle at home, recognised in town as a magician’s hex. She proposed to take him to a healer who could identify the evil influence and hopefully dispel it.

Before proceeding further with describing Ahmed’s predicament, it is necessary to provide some background information on magic and healing in the oasis. On an initial survey of the community, one would learn that the practices of magicians and healers are opposed; healers heal using the Qur’an (the word of God) and are on the side of good; magicians deploy the jinn (spirits of fire) through hexes and talismans to wreak havoc with a person and are on the side of evil. As I spent more time in Dakhla I realised that this neat distinction was not the whole truth. As far as people are concerned, and regardless of what a certain healer would claim, a common belief is that a very thin line separates healers from magicians and it is frequently crossed. This belief is supported by the following chain of thought: healers are human and cannot represent pure good which is something only Prophets are capable of. This renders them vulnerable to sin like everyone else, especially the sin of greed. By claiming to be healers people would trust them and seek them, yet by secretly engaging in magic they would compel people (through spells) to keep returning, thus making money off them on both counts. Given this widespread suspicion, it is natural that the people of Dakhla, who continue to take their afflictions to healers, are attuned to signs indicating that the healer they are now seeing also dabbles in magic.

After attending many healing sessions with various healers I found myself adept at detecting a presumed magician as some of the locals were. There is no written or agreed upon framework for making the distinction, and the key lies in subtle and highly subjective signs such as short, suspicious pauses during the Qur’anic reading. During these pauses, it is alleged, the healer/magician would slip the necessary talismans by silently invoking them or by making certain gestures with his fingers. Through my prolonged engagement with these sessions, gestures and movements which I might not have noticed before assumed a heightened salience for me. These otherwise meaningless movements engaged my attention and demanded interpretation.

Now Ahmed, at the suggestion of his family, consulted several healers. On one of these sessions, which was also to be the final one, things did not go very well. The man put up some incense as all healers do but while reading the specified Qur’anic verses the ashes fell to the ground and formed a square shape resembling the hex his mother had found; this seemed to Ahmed an incriminating sign. Two days later he found a dead cat at the family home, and drew a link between the death and the magician who had conducted the consultation in the same building. Combined, these two events left no doubt in Ahmed’s mind that the man really was a magician who was going to hurt rather than cure him. He refused to see him any further, and refused to see any other healers. Ahmed’s parents did not object to his refusal, in fact, they agreed with their son – as did their neighbours – that the man was probably a magician. When I asked why they thought so, they reiterated the common belief that most healers actually are; they do it to make more money but would never admit it. More specifically, they concurred with Ahmed that the similarity between the shape left by the ashes and the shape of the hex, and the death of the cat so soon after the healer’s visit, were evidence that that man was a magician.

Ahmed thus arrived at a consensual belief concerning the status of the healer who visited him. This belief was not only shared with his family and neighbours but also fitted in with the general mood in the community regarding healers/magicians. But how did he arrive at this belief? Two experiences and interpretations: the fallen ashes and the dead cat. With regards to the dead cat, it is a common belief that animals are sensitive to the presence of evil, sometimes to the point of death. That particular interpretation is quite straightforward. However, his interpretation of the fallen ashes is unusual, or, rather, not the interpretation as such but the very fact of taking the ashes as significant at all. As indicated above, and as I learnt through participation, what is significant or salient in the healing context with regards to the identification of magicians are pauses and gestures that indicate a talisman is being invoked. For Ahmed, however, the range of what is salient was broader and idiosyncratic, suggesting a deviation from culturally instilled experiential dispositions. This deviation constitutes a vulnerability as defined in the previous section, even if in Ahmed’s case others agreed with his interpretations. In fact, it is because of this agreement that we could say the vulnerability was masked by consensus in belief.^h^

### In the clinic

Having identified the vulnerability that lies at the heart of cultural (in)congruence, a vulnerability which cuts across social violations in norms, values and beliefs, the final question can now be addressed: is it possible to apply this richer sense of cultural congruence in clinical contexts? First, given the argument of this paper and the preceding case study, I will enumerate the different domains of knowledge and experience an observer is required to be familiar with in order to make cogent judgments of cultural (in)congruence:

(i) Identify a relevant sociocultural context.

(ii) Learn about this context: meanings and symbols which, in principle, others can tell us about, and perceptual alignment that can only be fully gained through participation.

(iii) Learn about the subject’s experiences and the foundation of her beliefs.

(iv) Apply the knowledge gained in (ii) in order to derive a view on the presence of otherwise of a vulnerability with the subject.

In the extended context of several months field-work and my extensive engagement with Ahmed, realising these four domains was achievable, enabling me to come up with the view explicated in the preceding case-study. This is clearly challenging in clinical contexts, where clinicians do not have the time, resources or expertise to apply cultural congruence in the way it has been expounded here. However, it may be possible to salvage something useful in terms of clinical applications if we carefully attend to the above domains.

First, in relation to identifying a relevant context, it should be clear that we are not after a label of so and so a culture or ethnicity whether this is assumed or provided by the individual. The assumption of bounded entities called cultures is untenable and cultural labels are at best heuristic devices that can serve as starting points for learning about a social context. Whether the subject is Pentecostal, Nubian, Maori or North-American while not irrelevant is not at all sufficiently instructive. The relevant social context is the *actual* context in which the individual lives and thrives and from where she draws, or is expected to draw, her fundamental interpretive frameworks and perceptual tendencies. In this sense, reducing a person’s sociocultural context to a label rather than exploring the intricacies of her or his actual environment is analogous to believing that if we wish to learn about a child’s home environment, all we need to do is consult local council statistics on employment and income in the neighbourhood. While we might learn something from the latter, it certainly is no substitute to contact with the child and the family.

Second, in relation to learning about this context, and given that it is highly unrealistic to expect clinicians to conduct fieldwork, there appears to be no solution here apart from seeking the best available knowledge but with a number of caveats in mind. The most obvious source of knowledge about the subject’s sociocultural context is his or her family members. Naturally they can provide valuable information both about the context as well as the subject. But it must be noted that this information is likely only to tackle symbolic dimensions of culture in addition to suffering with the problems of testimony such as bias, partial knowledge, expertise, and so on. Therefore, another important complementary source of knowledge is anthropologists with expertise on the community. Recent trends in anthropology favour phenomenological and what are called “experience-near” approaches to research. Works conducted in this methodological and theoretical framework, so long as they are current and relevant, may be particularly helpful since they go beyond culture as belief and attempt to explicate culture as a foundation of experience, a concern articulated in this paper and deemed important to a coherent concept of cultural congruence.

Part of the challenge here is to recognise that making judgements of (in)congruence requires access to domains of knowledge and expertise that lie outside the psychiatrist’s purview; appealing to other ‘experts’ is therefore unavoidable. However, this should be done with the above caveats in mind. For instance, appealing to a clinical department’s resident anthropologist or to the ‘Muslim Imam’ are helpful to the extent that either are deeply familiar with the subject’s actual sociocultural world, and are not just academically or externally acquainted as such.

Finally, assuming the first two issues have been addressed, domains three and four can proceed with relative ease. After all it is within a clinician’s remit and expertise to understand the subject’s experiences and enter her world.

## Conclusion

The psychiatric conceptualisation and application of cultural congruence may not help in detecting those who are vulnerable and may mislead into pathologising those who are not at risk. To address this problem, this paper sought to explore the concept, arguing that congruence should accommodate symbolic as well as phenomenological dimensions of culture. Given the view developed here, a richer sense of (in)congruence concerns alignment with or deviation from culturally instilled experiential dispositions which are foundational to a shared experience of the world. The problems of applying the concept in the richer sense articulated here have been noted. Clearly, from a practical point of view, the whole procedure will benefit if the clinical encounter is designed as a drawn-out and slowly unfolding process where knowledge of the sociocultural context is gained and developed one session after another as more and more people become involved and a genuine contextualisation of the subject’s experiences and beliefs is achieved – in a sense resembling ethnographic fieldwork. While this may not be ideal in clinical contexts where there is pressure to diagnose and treat, it might be essential for drawing a distinction between those cases where the subject is benignly expressing and realising a cultural script and others where this script is masking an unfolding vulnerability that might end up with that person on the margins of society.

## Endnotes

^a^Project website: http://www.dsm5.org/Pages/Default.aspx (accessed January 2013)

^b^Project website: http://transculturalpsychiatry.gla.ac.uk/ (accessed March 2013). Project was funded by the Arts and Humanities Research Council of England.

^c^In its original usage culture referred to activity; the “tending of natural growth” ([27], p. xvi). It denoted cultivating the land, breeding animals and looking “after one’s livelihood especially in its material aspects” ([8], p. 16). In Europe of the late middle-ages the locus of cultivation shifted and culture was identified with ‘civilisation’, denoting intellectual accomplishment and refinement and separating the European bourgeoisie from peasants and savages. In 18th and 19th century Europe, culture took an extended meaning when it began to be used as a noun in the context of developing nationalistic ideas that conceived of a nation as an exponent of a particular and unique world-view such as ‘Germanic Culture’ or ‘French Culture’. Immanuel Kant, in 1798, writes about the ‘character of the peoples’, describing the French as courteous, lovable and vivacious while the Englishman is self-sufficient, “claims only respect, and … wants only to live as he pleases” ([28], p. 214–217). The transformation of culture into a thing rather than an activity, and the association of this with what were thought of as higher values and accomplishments brought about discriminatory uses of culture. In 1869, Matthew Arnold was critical of the Victorian conception of culture which “plumes itself on a smattering of Greek and Latin” and is valued as “an engine of social and class distinction, separating its holder, like a badge or title, from other people who have not got it ([29], p.4). By the late 19th century the term ‘culture’ was taken into the academic discipline of anthropology. In *Primitive Culture,* Edward Tylor provided the first sufficiently abstract definition: “culture or civilisation .. is that complex whole which includes knowledge, belief, art, morals, law, custom, and any other capabilities and habits acquired by man as a member of a society”([30], p. 1). Here there is recognition of the universality of culture – everyone has culture – albeit within a paradigm of progression where ‘primitive culture’ was considered inferior to the rationally minded and technologically advanced ‘European culture’. Tylor operated within an evolutionary conception of civilisation where the ‘lower tribes’ were less developed than the ‘higher nations’.

^d^‘Capacity’ in this context refers to the ability to learn and apply shared frameworks of meaning and value.

^e^Cultural congruence in this sense would provide us with an insight into the moral and social registers of the mental states in question; whether a group of people think they are good, desirable, right, wrong or useful. These assessments are open to change and disagreement and are bound up with broader factors such as power and social relations. The question of the moral and social view of mental states is separate from the question of whether those mental states are significant, i.e. underpinned by a vulnerability, even if the answer to both questions may at times concur.

^f^This distinction does not depend on the presence of distress. If we were only after the alleviation of distress, we need not worry about cultural congruence, only about managing the distress. However, in seeking congruence we are concerned with subjects' embeddedness in their social environment, and this is of concern irrespective of distress. This embeddedness is characterised here in terms of partaking in shared perceptual alignments.

^g^Confidentiality and anonymity is upheld with regards to all study participants: no actual names are used and data that can aid identification has been omitted or changed. Ethical approval for the research on which this case study is based was obtained from University College London Research Ethics Committee (Project ID: 1521/001).

^h^In this case study, a vulnerability is masked by the presence of surface congruence in belief. Alternatively, the absence of surface congruence in belief need not imply that there is a vulnerability. Consider, for example, the case of a young man who joins a religious institution where the experiential dimensions of faith are encouraged. Members come to learn to attend to somatic sensations and thoughts and to re-interpret the origin and significance of these as the Spirit going through their body. The person’s mother and regular priest are concerned that this exceeds the bounds of normal experience and belief. In terms of the argument of this paper, there would be no vulnerability here. Through the religious sessions the man learnt to regard as salient aspects of experience that normally go unnoticed and to reinterpret those through a new framework.

## Competing interests

The authors declare that they have no competing interest.
